# High-Resolution MRI-Based Clinical–Radiological Phenotypes of Carotid Vulnerability and Long-Term Outcomes After Carotid Revascularization: A Single-Center Retrospective Cohort Study

**DOI:** 10.3390/jcm15155884

**Published:** 2026-07-28

**Authors:** Sunan Xu, Yunhao Lei, Tingting Li, Yumeng Sun, Zhenjia Wang, Wei Yu

**Affiliations:** Department of Radiology, Beijing Anzhen Hospital, Capital Medical University, 2 Anzhen Road, Chaoyang District, Beijing 100029, China; drxusunan@mail.ccmu.edu.cn (S.X.);

**Keywords:** carotid atherosclerosis, high-resolution magnetic resonance imaging, plaque vulnerability, latent class analysis, major adverse cardiovascular events

## Abstract

**Background/Objectives**: Patients undergoing carotid revascularization may show distinct patterns of bilateral carotid vulnerability and clinical risk. This study used latent class analysis (LCA) to identify high-resolution magnetic resonance imaging (HR-MRI)-based clinical–radiological phenotypes and evaluate their association with long-term major adverse cardiovascular events (MACE). **Methods**: This retrospective cohort included 289 patients who underwent carotid endarterectomy (CEA) or carotid artery stenting (CAS) between April 2017 and April 2024 and had preoperative carotid HR-MRI within 90 days. Ipsilateral and contralateral plaque features were assessed on multi-contrast vessel-wall HR-MRI. LCA included five indicators: ipsilateral intraplaque hemorrhage, ipsilateral lipid-rich necrotic core, contralateral intraplaque hemorrhage, cardiovascular disease history, and symptomatic status. MACE comprised cardiovascular death, nonfatal myocardial infarction, coronary revascularization, or stroke. Outcomes were assessed using Kaplan–Meier analysis and multivariable Cox regression. **Results**: Among 289 patients undergoing carotid revascularization, the mean age was 65.12 ± 9.46 years, and 237 (82.0%) patients were men. LCA identified three phenotypes: Systemic High-Risk (*n* = 128), Silent High-Risk (*n* = 37), and Stable/Moderate (*n* = 124). The Systemic High-Risk phenotype showed high probabilities of symptomatic presentation, cardiovascular disease history, and bilateral carotid vulnerability, whereas the Silent High-Risk phenotype showed marked ipsilateral plaque vulnerability despite lower clinical risk. During a median follow-up of 4.4 years, MACE-free survival differed significantly across phenotypes (log-rank *p* = 0.043). Compared with the Stable/Moderate phenotype, the Systemic High-Risk and Silent High-Risk phenotypes were both associated with increased MACE risk (HR 1.65, 95% CI 1.15–2.37, *p* = 0.039; HR 1.43, 95% CI 1.12–2.15, *p* = 0.034). **Conclusions**: HR-MRI-based LCA identified clinically meaningful carotid vulnerability phenotypes associated with long-term MACE after carotid revascularization. These findings support integrated assessment of bilateral plaque vulnerability and clinical risk for postoperative cardiovascular risk stratification.

## 1. Introduction

Carotid atherosclerosis is an important source of ischemic cerebrovascular events, including stroke and transient ischemic attack (TIA), which remain leading causes of death and disability worldwide [1]. Although luminal stenosis has historically guided clinical decision making for carotid intervention, it does not fully capture the heterogeneous biological behavior of atherosclerotic plaques. Vulnerable plaque features on magnetic resonance imaging (MRI), such as intraplaque hemorrhage (IPH), lipid-rich necrotic core (LRNC), and fibrous cap thinning/rupture, have been strongly associated with subsequent ischemic events independent of stenosis severity [2,3,4]. Carotid high-resolution MRI (HR-MRI), particularly black-blood techniques, enables direct visualization of the arterial wall and plaque components beyond luminal narrowing by suppressing the luminal blood signal and improving vessel-wall contrast. This approach facilitates the identification of vulnerable plaque features and may provide incremental information for ischemic stroke risk assessment beyond conventional stenosis-based evaluation [2,3,4]. Meta-analytic evidence further confirms MRI-detected IPH and LRNC substantially increase the risk of future cerebrovascular events, underscoring the added value of plaque composition imaging over conventional angiographic metrics [5,6].

However, most previous studies have evaluated plaque vulnerability using individual imaging markers or focused primarily on the ipsilateral culprit lesion. This feature-based approach may not fully capture the multidimensional nature of vascular risk in patients undergoing carotid revascularization, in whom long-term outcomes may be shaped by both local plaque vulnerability and broader systemic vascular risk. Recent evidence has emphasized a shift from stenosis-centered assessment toward more comprehensive risk stratification incorporating plaque morphology, plaque composition and individualized clinical risk profiles [7]. Similarly, MRI-based risk models integrating plaque vulnerability features with clinical indicators have shown promise for individualized stroke risk assessment in symptomatic carotid artery disease [8]. Therefore, an integrated phenotype-based framework combining HR-MRI-derived plaque features with clinically relevant risk indicators may provide a more comprehensive understanding of carotid vulnerability and its prognostic implications after revascularization.

Latent class analysis (LCA) is a data-driven approach that identifies unobserved subgroups within heterogeneous populations by integrating multiple interrelated indicators. LCA has been successfully applied in cardiovascular and other clinical studies to define phenotypic clusters with distinct risk profiles and prognostic relevance [9,10]. In the context of carotid atherosclerosis, LCA may help move beyond isolated feature-based interpretation and uncover clinically meaningful vulnerability phenotypes reflecting different patterns of local plaque instability and systemic vascular risk.

In this study, we applied LCA to HR MRI-based plaque characteristics from both ipsilateral and contralateral carotid arteries in patients undergoing revascularization, with the aims to (i) define distinct carotid vulnerability phenotypes based on multiple imaging and clinical data, (ii) characterize their clinical and imaging correlates, and (iii) explore associations with major adverse cardiovascular events (MACE). We hypothesized that data-driven vulnerability phenotypes would more effectively stratify patients than traditional measures alone, providing a framework for refined risk assessment and personalized clinical management.

## 2. Materials and Methods

### 2.1. Study Design and Population

This single-center retrospective cohort study was conducted at a tertiary cardiovascular center and approved by the Institutional Review Board (Approval No. 2025265x). Written informed consent was waived. The study was conducted in accordance with the Declaration of Helsinki.

**Figure 1:**
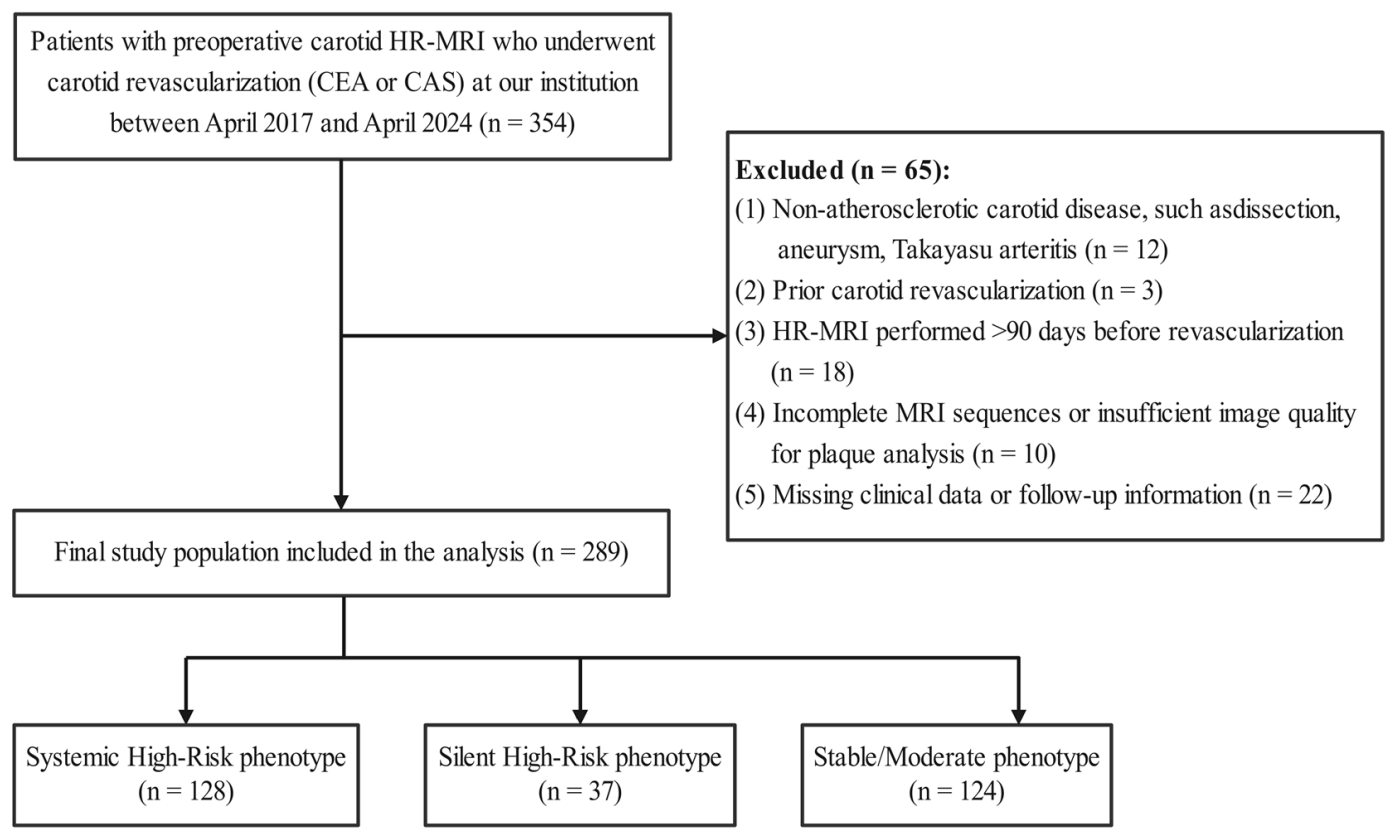
Patient selection flowchart.

At our center, carotid HR-MRI is frequently performed as part of the preoperative imaging evaluation for patients considered for carotid revascularization, when clinically feasible and in the absence of MRI contraindications. A total of 354 patients with preoperative carotid HR-MRI who underwent carotid revascularization, including carotid endarterectomy (CEA) or carotid artery stenting (CAS), at our institution between April 2017 and April 2024 were retrospectively screened. Patients were excluded for any of the following reasons: (1) non-atherosclerotic carotid disease, such as dissection, aneurysm, or Takayasu arteritis (*n* = 12); (2) prior ipsilateral or contralateral carotid revascularization (*n* = 3); (3) HR-MRI performed more than 90 days before revascularization (*n* = 18); (4) incomplete MRI sequences or insufficient image quality for reliable plaque characterization (*n* = 10); or (5) missing clinical data or missing follow-up information (*n* = 22). Incomplete MRI sequences were defined as the absence of one or more sequences required for reliable plaque characterization, including 3D TOF MRA, black-blood T1-weighted imaging, black-blood T2-weighted imaging, 3D SNAP, or contrast-enhanced T1-weighted imaging. This situation could occur when patients were unable to tolerate the full MRI examination because of the relatively long scan duration, resulting in early termination of the MRI protocol. Missing clinical data were defined as missing variables required for phenotype construction or adjusted outcome analysis, including symptomatic status, cardiovascular disease history, age, sex, and procedure type. A total of 289 patients were included in the final analysis. The patient selection process is shown in Figure 1.

### 2.2. Clinical and Laboratory Data Collection

Baseline demographic and clinical data were extracted from electronic medical records, including age, sex, body mass index (BMI), symptom status, hypertension, diabetes mellitus, hyperlipidemia, cardiovascular disease (CVD) history and procedure type. BMI was calculated as body weight divided by height squared (kg/m^2^). Symptom status was defined as ipsilateral neurological symptoms attributable to carotid atherosclerotic disease, including ischemic stroke, transient ischemic attack, or amaurosis fugax [11].

Hypertension was defined as a history of hypertension, current use of antihypertensive medication, or repeatedly elevated blood pressure [12]. Diabetes mellitus was defined as a history of diabetes, current use of glucose-lowering medication, or elevated fasting plasma glucose [13]. Hyperlipidemia was defined as a history of dyslipidemia, current use of lipid-lowering therapy, or abnormal lipid profiles recorded before revascularization [14]. CVD history was defined as a documented history of clinically established cardiovascular or cerebrovascular disease, including coronary artery disease, myocardial infarction, coronary revascularization, ischemic stroke, or transient ischemic attack.

Preoperative lipid profiles, including total cholesterol (TC), high-density lipoprotein cholesterol (HDL-c), low-density lipoprotein cholesterol (LDL-c), and triglycerides (TG), were obtained. When multiple laboratory measurements were available, the value closest to the date of revascularization was used for analysis.

### 2.3. HR-MRI Acquisition and Plaque Assessment

Preoperative carotid plaque imaging was performed using a 3.0-T MR scanner (Ingenia CX, Philips Healthcare, Best, The Netherlands) with a 32-channel head coil and an 8-channel dedicated neck coil. Imaging covered both carotid bifurcations, centering at the site of maximal plaque burden. A standardized multi-contrast vessel-wall imaging protocol was applied, including 3D time-of-flight (TOF), black-blood T1- and T2-weighted sequences, 3D SNAP (simultaneous non-contrast angiography and intraplaque hemorrhage), and contrast-enhanced T1-weighted imaging. Detailed acquisition parameters for each sequence are provided in Table 1. Image quality was assessed using a four-point scale [15]. Examinations were considered of insufficient quality when severe motion artifacts, poor signal-to-noise ratio, inadequate blood suppression, incomplete anatomical coverage, or other artifacts prevented reliable delineation of the lumen and outer wall boundaries or reliable assessment of plaque components. Such examinations were excluded from the final analysis.

The treated carotid artery was defined as the ipsilateral side, and the opposite artery was defined as the contralateral side. Ipsilateral plaque burden was quantified using dedicated vessel-wall analysis software (Vessel Explorer 2.0, TSImaging Healthcare, Beijing, China). Lumen and outer wall boundaries were manually delineated on cross-sectional vessel-wall images. The normalized wall index (NWI) was calculated as: NWI = wall area/total vessel area × 100%, where total vessel area was defined as the area enclosed by the outer wall boundary.

**Figure 2:**
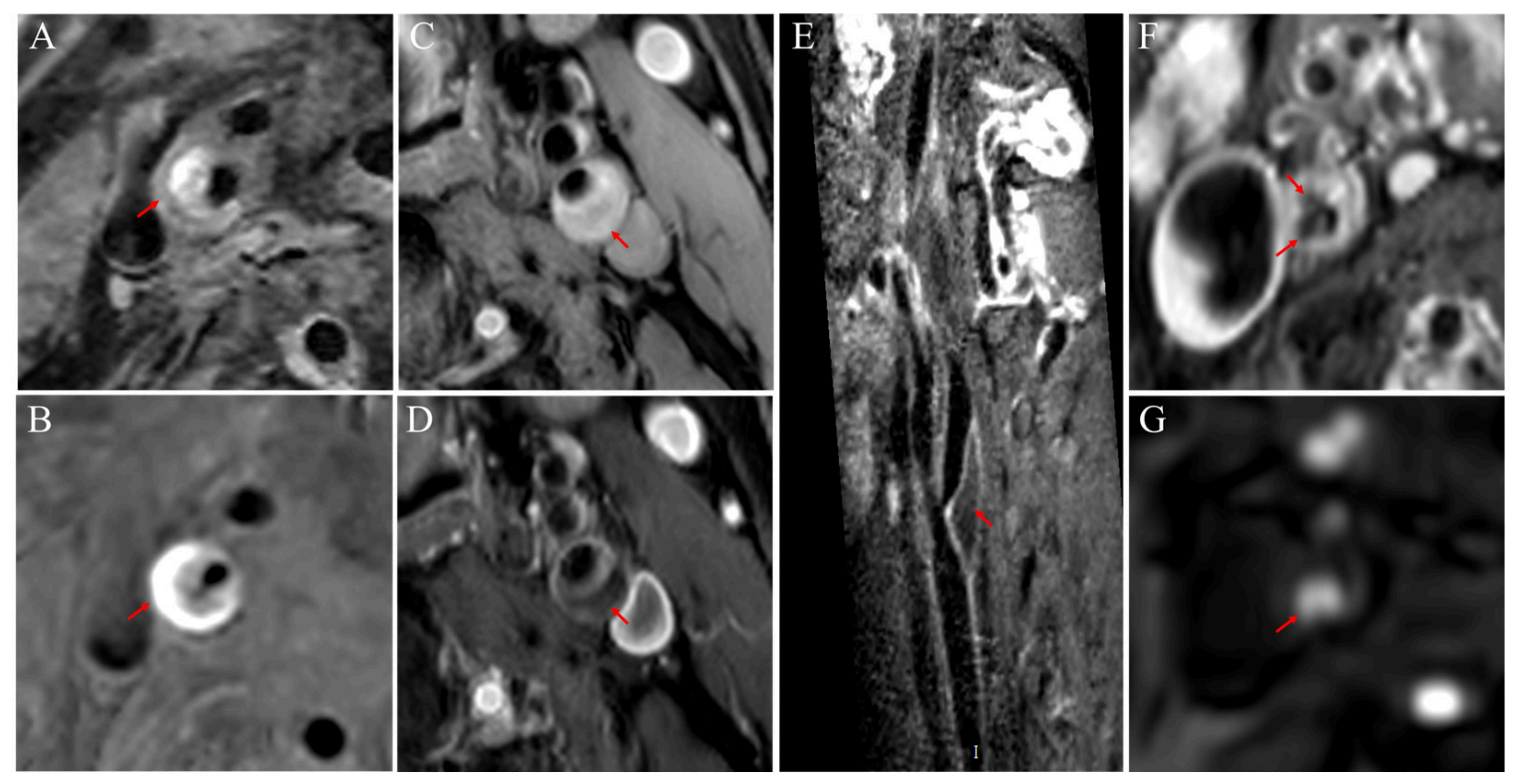
Representative HR-MRI findings of carotid plaque vulnerability. (**A**,**B**) Axial non-contrast T1-weighted image and simultaneous non-contrast angiography and intraplaque hemorrhage (SNAP) image showing intraplaque hemorrhage (IPH). (**C**–**E**) Axial non-contrast T1-weighted image, axial contrast-enhanced T1-weighted image, and sagittal contrast-enhanced T1-weighted image showing lipid-rich necrotic core (LRNC). (**F**,**G**) Contrast-enhanced T1-weighted image and time-of-flight (TOF) MR angiography showing plaque ulceration. Red arrows indicate representative plaque vulnerability features.

Ipsilateral plaque components, including IPH, LRNC and plaque ulceration, were assessed qualitatively and recorded as present or absent according to established multi-contrast MRI criteria [16]. The ability of carotid HR-MRI to characterize major plaque components, particularly IPH and LRNC, has been validated against histopathological findings from carotid endarterectomy specimens in previous studies (Supplementary Table S1). Representative HR-MRI findings of IPH, LRNC, and plaque ulceration are shown in Figure 2. Contralateral plaque features were evaluated using the same criteria. Contralateral luminal stenosis was categorized as mild (<50%) or moderate-to-severe (≥50%) according to the North American Symptomatic Carotid Endarterectomy Trial (NASCET) criteria [17].

All images were independently reviewed by two radiologists with at least 3 years of experience in vascular imaging, who were blinded to clinical data, treatment information, and follow-up outcomes. Disagreements were resolved by consensus, with adjudication by a senior radiologist when necessary. Interobserver reproducibility was assessed in a randomly selected subset of 30 patients using the intraclass correlation coefficient (ICC) for NWI and Cohen’s κ statistics for qualitative plaque features.

### 2.4. MRI Vulnerability Score Construction

Separate MRI vulnerability scores were constructed to summarize the burden of high-risk plaque features in the ipsilateral and contralateral carotid arteries. The ipsilateral score was designed to reflect culprit plaque vulnerability and included established high-risk plaque features, including IPH, LRNC, plaque ulceration, and increased plaque burden defined as NWI > 70% [18]. The contralateral score was designed to reflect the systemic burden of carotid atherosclerosis and included contralateral IPH, LRNC, ulceration, and moderate-to-severe stenosis, defined as stenosis ≥ 50%.

Each feature was assigned 1 point when present. The total ipsilateral and contralateral vulnerability scores were calculated by summing the number of present features, yielding a score range of 0 to 4 for each side. Higher scores indicated greater culprit plaque vulnerability for the ipsilateral artery and greater carotid atherosclerotic burden for the contralateral artery.

### 2.5. Follow-Up and Outcomes

Follow-up started from the date of carotid revascularization. Patients were followed through outpatient visits, medical records review and telephone interviews. Follow-up was updated around October 2025. Patients without any postprocedural follow-up information were excluded. For patients without MACE, at least 12 months of follow-up after carotid revascularization was required. Patients who experienced MACE within 12 months were retained, and the time to the first event was recorded.

The primary outcome was major adverse cardiovascular events (MACE), defined as the first occurrence of cardiovascular death, nonfatal myocardial infarction, coronary revascularization, or stroke. For each patient, the occurrence and timing of the first MACE were recorded. Patients without MACE were censored at the date of last available follow-up.

### 2.6. Latent Class Analysis

Latent class analysis (LCA) was performed to identify distinct clinical–radiological carotid vulnerability phenotypes integrating culprit plaque vulnerability, systemic atherosclerotic burden, and clinical presentation. Five binary indicators were selected for LCA: ipsilateral intraplaque hemorrhage (IPH), ipsilateral lipid-rich necrotic core (LRNC), contralateral IPH, history of cardiovascular disease (CVD), and symptomatic status before revascularization. Ipsilateral IPH and LRNC were included to represent high-risk features of the treated culprit plaque, whereas contralateral IPH and CVD history were included to reflect systemic atherosclerotic vulnerability. Symptomatic status was included to capture the clinical manifestation of carotid atherosclerotic disease and to integrate clinical presentation with HR-MRI-derived plaque vulnerability features. These indicators were selected a priori to represent culprit plaque vulnerability, systemic atherosclerotic vulnerability, and clinical presentation, while maintaining the clinical interpretability of the latent class model.

LCA models with different numbers of classes were fitted using maximum likelihood estimation. Model selection was based on the Akaike information criterion (AIC) and Bayesian information criterion (BIC), together with class size, posterior classification probability, and clinical interpretability [19,20]. Patients were assigned to the latent class with the highest posterior probability. The latent classes were subsequently labelled according to their conditional probability profiles as Systemic High-Risk, Silent High-Risk, and Stable/Moderate phenotypes.

### 2.7. Statistical Analysis

Continuous variables are presented as mean ± standard deviation or median with interquartile range, as appropriate. Categorical variables are presented as counts and percentages.

Baseline demographic, clinical, laboratory, and imaging characteristics were compared across LCA-derived phenotypes. One-way analysis of variance or the Kruskal–Wallis test was used for continuous variables, whereas the χ^2^ test or Fisher’s exact test was used for categorical variables, as appropriate.

Time-to-event outcomes were evaluated using Kaplan–Meier survival curves and compared using the log-rank test. Cox proportional hazards models were used to estimate hazard ratios (HRs) and 95% confidence intervals (CIs) for the association between LCA-derived carotid vulnerability phenotypes and MACE. The Stable/Moderate phenotype was used as the reference group. The primary multivariable model was adjusted for clinically relevant covariates, including age, sex and procedure type. The proportional hazards assumption was assessed using Schoenfeld residuals. All statistical analyses were performed using R software version 4.2.0 (R Foundation for Statistical Computing, Vienna, Austria). All statistical tests were two-sided, and *p* < 0.05 was considered statistically significant.

## 3. Results

### 3.1. Clinical and Imaging Characteristics

Baseline clinical and imaging characteristics are shown in Table 2. Among the 289 patients, Class 1, class 2, and class 3 included 128, 37 and 124 patients, respectively. The three classes differed significantly in symptom status, age, sex distribution, CVD history, procedure type, and plaque vulnerability features. Symptomatic presentation was present in all patients in class 1, absent in Class 2, and observed in 71.0% of patients in Class 3 (*p* < 0.001), consistent with the symptomatic and silent profiles of the identified phenotypes. Age also differed across classes, with Class 1 and Class 2 being older than Class 3 overall (66.8 ± 7.9 and 65.5 ± 7.1 years vs. 63.3 ± 11.1 years, *p* = 0.014). Class 1 showed the highest prevalence of CVD history compared with Class 2 and Class 3 (56.2% vs. 29.7% and 38.7%, *p* = 0.002), whereas Class 2 had the highest proportion of male patients (91.9% vs. 88.3% and 72.6%, *p* = 0.001). Procedure type also differed significantly across classes, with CEA more frequently performed in Class 1 (62.5%) and class 2 (59.5%) and CAS more frequently performed in Class 3 (62.1%) (*p* < 0.001). Other conventional risk factors were generally comparable among the three classes, including BMI, hypertension, diabetes, hyperlipidemia, and lipid profiles (all *p* > 0.05).

Marked differences were observed in bilateral plaque vulnerability features. Ipsilateral IPH was present in 85.2% of Class 1 and 100.0% of Class 2, but was absent in Class 3 (*p* < 0.001). Ipsilateral LRNC was highly prevalent across all classes, although the interclass difference remained statistically significant (*p* = 0.017). Contralateral IPH showed a similar pattern, being more frequent in Class 1 and Class 2 than in Class 3 (45.3% and 40.5% vs. 2.4%, *p* < 0.001). Contralateral LRNC and moderate-to-severe stenosis also differed significantly across classes (both *p* ≤ 0.001), supporting a greater bilateral plaque burden in the high-risk phenotypes. In contrast, plaque ulceration did not differ significantly among classes. Overall, the LCA-derived phenotypes were mainly distinguished by symptomatic status, CVD history and bilateral plaque vulnerability features, whereas most conventional cardiovascular risk factors were similar across groups. Interobserver reproducibility was good to excellent in the subset of 30 randomly selected patients, with an ICC of 0.82 for NWI and κ values of 0.90, 0.88, and 0.82 for IPH, LRNC, and ulceration, respectively.

### 3.2. Latent Class Analysis and Phenotype Profiles

**Figure 3:**
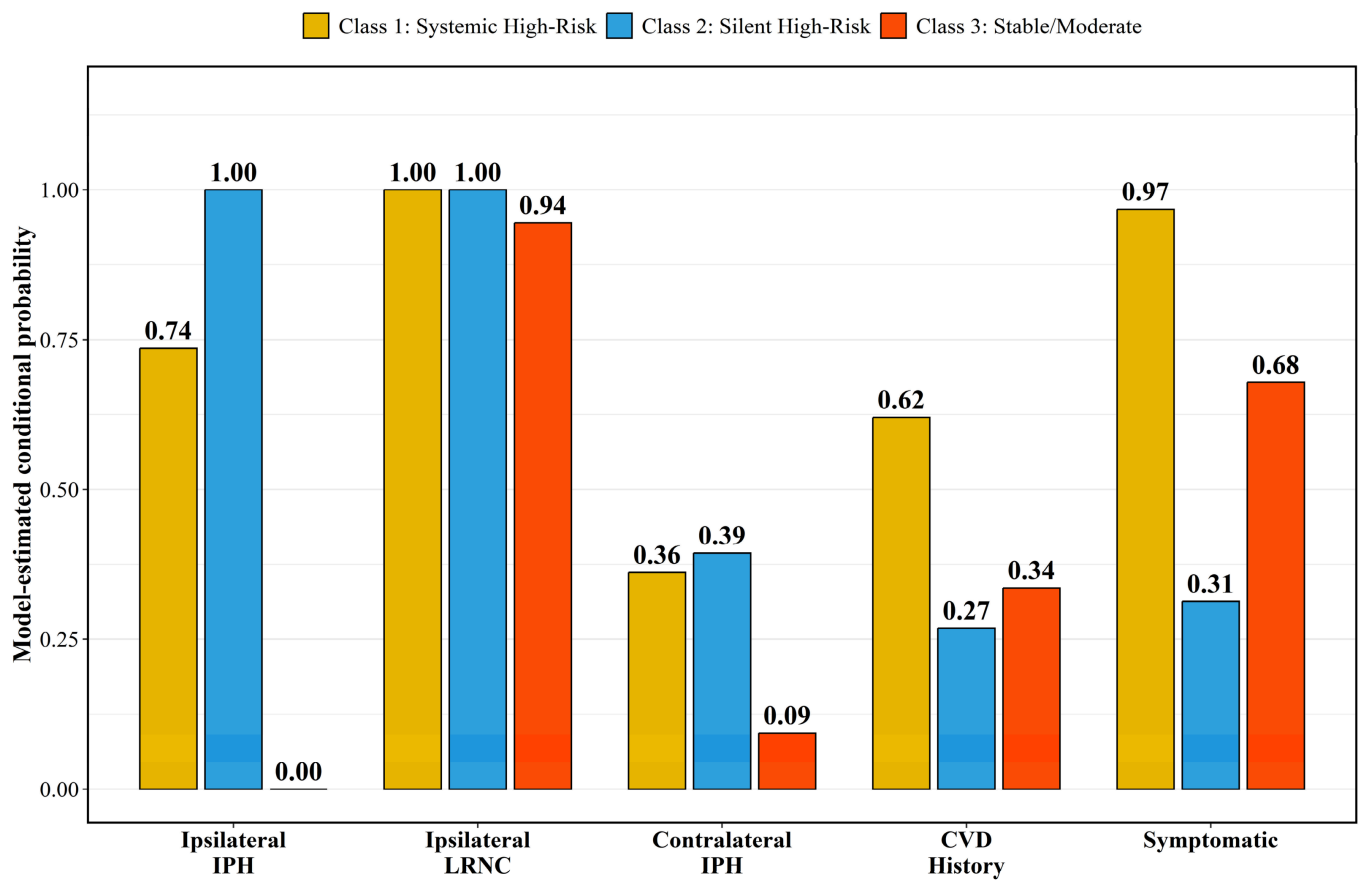
Latent class profiles of HR-MRI–based carotid vulnerability phenotypes. IPH, intraplaque hemorrhage; LRNC, lipid-rich necrotic core; CVD, cardiovascular disease.

Latent class analysis identified three distinct carotid vulnerability phenotypes based on HR-MRI plaque features and clinical indicators (Figure 3). Class 1, defined as Systemic High-Risk phenotype (44.3%), was characterized by high probabilities of ipsilateral IPH, ipsilateral LRNC, CVD history and symptomatic presentation, suggesting combined culprit plaque vulnerability and systemic vascular risk. Class 2, defined as Silent High-Risk phenotype (12.8%), showed uniformly high probabilities of ipsilateral IPH and LRNC, but relatively low probabilities of symptomatic presentation and CVD history, indicating marked ipsilateral plaque vulnerability despite a clinically silent profile. Class 3, defined as Stable/Moderate phenotype (42.9%), was characterized by the absence of ipsilateral IPH, a low probability of contralateral IPH, and an intermediate probability of symptomatic presentation. Overall, the three latent classes were mainly differentiated by IPH distribution, CVD history, and symptomatic status, whereas ipsilateral LRNC showed high probabilities across all classes.

### 3.3. Bilateral MRI Vulnerability Scores Across Latent Phenotypes

**Figure 4:**
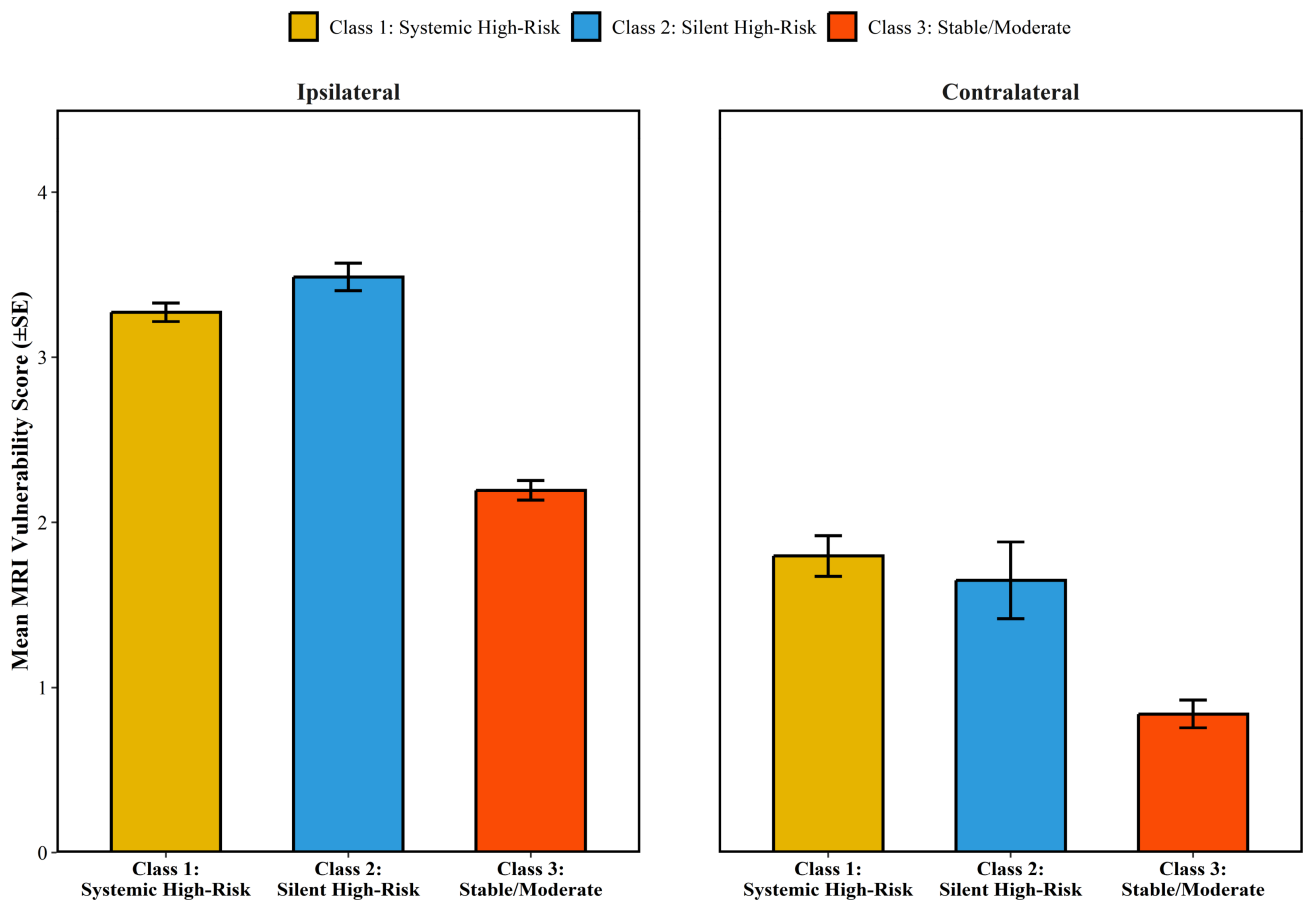
Bilateral MRI vulnerability scores across HR-MRI–based carotid vulnerability phenotypes.

To further quantify bilateral plaque vulnerability, ipsilateral and contralateral MRI vulnerability scores were compared across the three latent phenotypes (Figure 4). In all three classes, ipsilateral carotid arteries showed significantly higher vulnerability scores than contralateral arteries. The mean ipsilateral versus contralateral scores were 3.27 versus 1.80 in Class 1, 3.49 versus 1.65 in Class 2, and 2.19 versus 0.84 in Class 3, respectively, with significant within-patient side differences in each class (all *p* < 0.001).

In the linear mixed-effects model accounting for within-patient bilateral measurements, ipsilateral side remained strongly associated with a higher MRI vulnerability score after adjustment for age, sex, symptom status, and CVD history (β = 1.48, 95% CI: 1.33–1.63, *p* < 0.001). Contralateral vulnerability scores were higher in Class 1 and Class 2 than in Class 3 (1.80 and 1.65 vs. 0.84), consistent with the patterns observed in the latent class profiles.

### 3.4. Major Adverse Cardiovascular Event Across Latent Phenotypes

During a median follow-up of 4.4 years, a total of 66 (22.8%) major adverse cardiovascular events (MACE) were recorded among 289 patients. The incidence of MACE was 25.0%, 24.3%, and 20.2% in Class 1 (Systemic High-Risk), Class 2 (Silent High-Risk), and Class 3 (Stable/Moderate), respectively.

**Figure 5:**
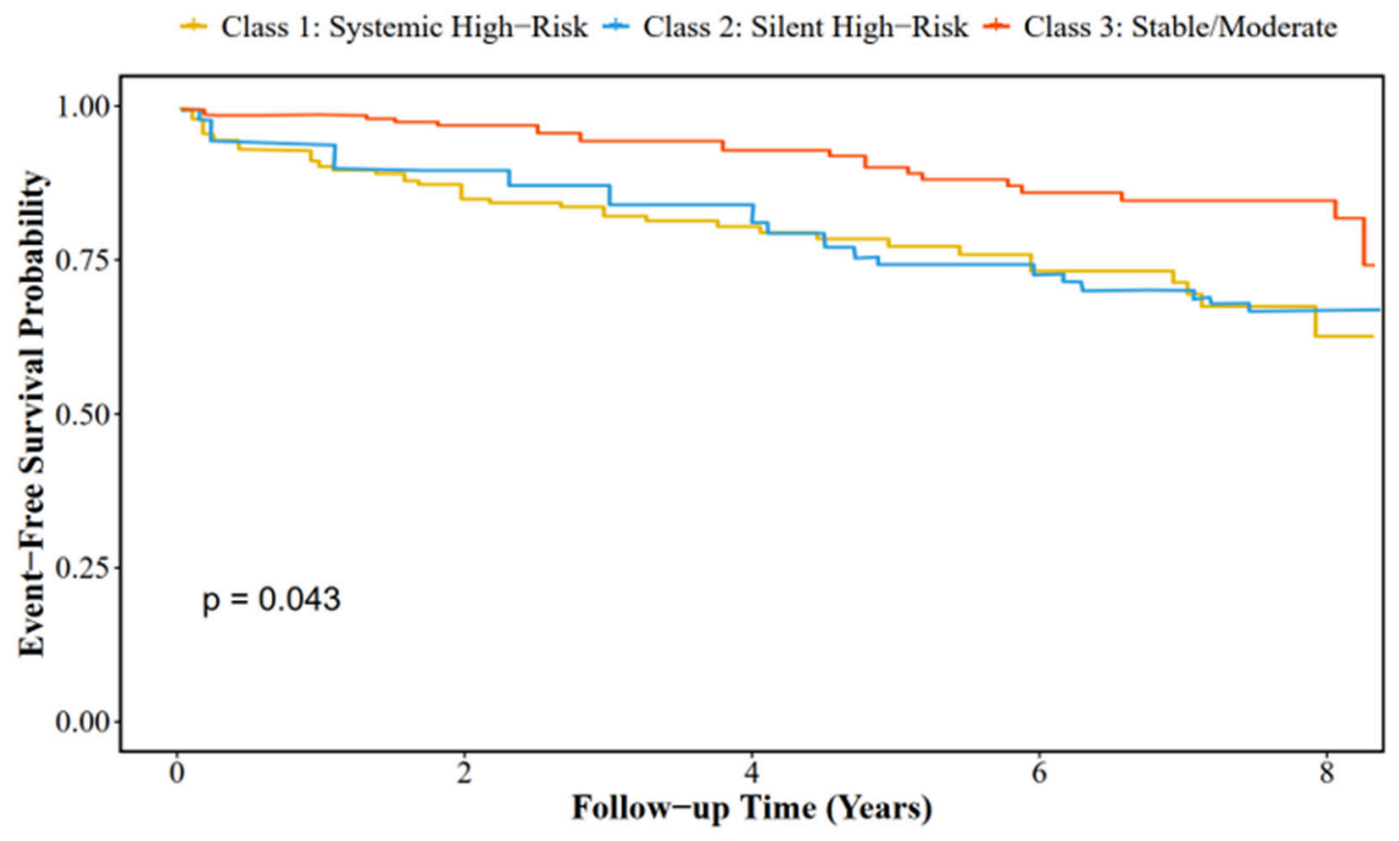
Kaplan–Meier curves for MACE-free survival stratified by latent phenotypes.

Kaplan–Meier survival analysis showed a separation of MACE-free survival curves across the three latent classes (log-rank *p* = 0.034; Figure 5). In multivariable Cox regression adjusting for age, sex and procedure type, both Systemic High-Risk phenotype and Silent High-Risk phenotype were associated with an increased risk of MACE compared with the Stable/Moderate phenotype (Class 1: HR 1.65, 95% CI 1.15–2.37, *p* = 0.039; Class 2: HR 1.43, 95% CI 1.12–2.15, *p* = 0.034) (Table 3).

## 4. Discussion

In this retrospective cohort of patients undergoing carotid revascularization, we identified three distinct clinical–radiological carotid vulnerability phenotypes using latent class analysis. The Systemic High-Risk phenotype was characterized by a high probability of symptomatic presentation, frequent cardiovascular disease history, and increased ipsilateral and contralateral vulnerability burden. The Silent High-Risk phenotype showed marked ipsilateral plaque vulnerability, characterized by high probabilities of ipsilateral IPH and LRNC, but relatively low probabilities of symptomatic presentation and CVD history. In contrast, the Stable/Moderate phenotype showed lower probabilities of IPH and bilateral MRI vulnerability scores. Importantly, both high-risk phenotypes were associated with worse MACE-free survival compared with the Stable/Moderate phenotype, suggesting that integrated assessment of plaque vulnerability and clinical risk status may provide clinically relevant prognostic information after carotid revascularization.

The understanding of carotid artery disease has evolved from a primarily stenosis-centered model toward a broader concept incorporating plaque composition, plaque instability, local hemodynamic factors, inflammation, and systemic atherosclerotic risk [7]. From a pathological perspective, atherosclerotic plaques undergo progressive evolution from early lesions to advanced complicated plaques, with features such as lipid accumulation, necrotic core formation, fibrous cap disruption, and intraplaque hemorrhage representing important stages of plaque instability described in the AHA histopathological classification system [21]. Although luminal stenosis remains central to clinical decision-making, plaque morphology and biological activity may better reflect the risk of plaque destabilization and downstream vascular events. In this context, the LCA-derived phenotypes in the present study support the concept that carotid vulnerability should be interpreted as a combined clinical–radiological construct rather than as an isolated single imaging marker.

A major finding of this study is that carotid vulnerability in patients undergoing revascularization cannot be fully captured by symptom status or individual plaque features alone. The present findings further support the value of carotid HR-MRI for evaluating plaque vulnerability in patients undergoing carotid revascularization. In this study, multi-contrast HR-MRI allowed assessment of both ipsilateral and contralateral plaque features, including IPH, LRNC, ulceration, and plaque burden, which may help identify patients with biologically unstable plaques and increased long-term cerebrovascular or systemic cardiovascular risk [16,22]. Carotid ultrasound remains an important first-line imaging modality for evaluating carotid stenosis, flow velocity, and general plaque morphology. However, routine ultrasound mainly provides luminal and hemodynamic information and has limited ability to directly and comprehensively characterize plaque tissue components, particularly IPH and LRNC, in a standardized component-based manner. In contrast, multi-contrast carotid HR-MRI enables direct visualization of the vessel wall and plaque composition, allowing bilateral assessment of plaque vulnerability based on IPH, LRNC, plaque morphology, and plaque burden. Therefore, HR-MRI provides incremental information beyond conventional ultrasound and was essential for constructing the clinical–radiological phenotypes in the present study. Contrast-enhanced ultrasound (CEUS) has also been used to evaluate carotid plaque vulnerability by detecting intraplaque neovascularization and enhancement patterns associated with plaque inflammation and biological activity. Compared with CEUS, HR-MRI provides more detailed characterization of plaque composition, particularly IPH and LRNC. These imaging modalities may offer complementary information for comprehensive assessment of carotid plaque vulnerability [23]. In addition to noninvasive vascular imaging techniques, invasive modalities such as optical coherence tomography (OCT) have provided detailed insights into the microstructural characteristics of vulnerable plaques, including fibrous cap disruption, lipid accumulation, and inflammatory cell infiltration, particularly in coronary arteries [24]. However, OCT is not routinely applicable for carotid plaque assessment because of its invasive nature and limited feasibility in extracoronary vascular beds. In contrast, carotid HR-MRI enables noninvasive evaluation of plaque composition and vulnerability features in vivo.

Recent evidence has increasingly emphasized that risk stratification in carotid atherosclerosis should extend beyond luminal stenosis to incorporate plaque composition and morphology, including IPH, LRNC, and plaque surface disruption, which are established markers of plaque vulnerability [3,22,25]. Previous studies have further demonstrated that HR-MRI-derived plaque characteristics, particularly IPH and LRNC, are associated with future cerebrovascular and cardiovascular events after carotid revascularization, supporting their prognostic value beyond conventional clinical and luminal imaging parameters [3,4,5,6]. In the present study, we further extended this concept by integrating multiple plaque features and clinical indicators through LCA to identify distinct vulnerability phenotypes associated with long-term MACE. Previous studies using high-resolution black-blood carotid MRI have demonstrated that patients with recent ischemic stroke frequently exhibit vulnerable plaque characteristics, including IPH, LRNC, and fibrous cap disruption, supporting the association between vessel-wall MRI findings and symptomatic cerebrovascular events [2,22]. In particular, IPH has been consistently associated with increased risk of future ischemic events in both symptomatic and asymptomatic carotid stenosis [3]. Our Silent High-Risk phenotype supports this concept from another perspective: despite a lower probability of symptomatic presentation, this subgroup showed substantial ipsilateral plaque vulnerability and poorer event-free survival than the Stable/Moderate phenotype. This finding suggests that clinically silent patients may still harbor biologically active and prognostically relevant plaque features.

The Systemic High-Risk phenotype may represent a different but equally important risk pathway. Compared with the Silent High-Risk phenotype, this class was characterized not only by high ipsilateral vulnerability but also by a higher probability of CVD history and greater contralateral plaque burden. Atherosclerosis is a systemic inflammatory and lipid-driven disease that affects multiple vascular territories, and clinical events after carotid revascularization may arise from both local carotid mechanisms and broader systemic vascular vulnerability [26,27]. Therefore, the increased MACE risk observed in this phenotype may reflect the combined effect of culprit plaque vulnerability and systemic atherosclerotic burden. This is clinically meaningful because revascularization addresses the treated carotid lesion but does not eliminate the patient’s underlying systemic vascular risk.

Our findings also highlight the value of a phenotype-based approach. Traditional risk stratification in carotid disease has largely relied on stenosis severity, symptom status, and selected clinical risk factors, while contemporary evidence increasingly supports the role of plaque vulnerability imaging in refining risk assessment [11,16,28]. However, individual imaging markers are often correlated and may represent overlapping biological processes. LCA provides a data-driven framework to integrate multiple interrelated indicators and identify clinically interpretable subgroups that may not be apparent from isolated variable-by-variable analyses [20,29]. In this study, the distinction between the Systemic High-Risk and Silent High-Risk phenotypes illustrates this advantage: both classes showed high plaque vulnerability, but they differed in clinical presentation and systemic disease burden, suggesting potentially different mechanisms of long-term risk.

The prognostic separation observed in the Kaplan–Meier analysis further supports the clinical relevance of these phenotypes. Compared with the Stable/Moderate phenotype, both the Systemic High-Risk and Silent High-Risk phenotypes showed lower MACE-free survival. Multivariable Cox analysis showed that these high-risk phenotypes remained associated with increased MACE risk after adjustment for age, sex and procedure type. These findings are consistent with recent evidence showing that MRI-defined carotid plaque composition, particularly IPH and LRNC, is associated with future cerebrovascular and cardiovascular events beyond conventional clinical risk factors and luminal stenosis [30,31,32]. Therefore, the LCA-derived phenotypes may capture a cumulative vulnerability burden that is not fully reflected by any single plaque feature or clinical variable.

The identification of a Silent High-Risk phenotype may have particular clinical implications. Contemporary evidence on asymptomatic carotid stenosis increasingly emphasizes that the absence of recent neurological symptoms does not necessarily indicate biological plaque stability, and imaging markers such as IPH can help identify patients at higher stroke risk despite an asymptomatic presentation [33]. Our results extend this concept to patients undergoing carotid revascularization by suggesting that clinically silent but plaque-vulnerable patients may still have an unfavorable long-term risk profile. Such patients may require closer post-procedural surveillance and more intensive secondary prevention, particularly when HR-MRI demonstrates IPH and LRNC. Conversely, patients in the Systemic High-Risk phenotype may benefit from a broader cardiovascular risk management, including optimization of lipid-lowering therapy, antithrombotic therapy when appropriate, blood pressure and glucose control, and assessment of concomitant coronary or peripheral atherosclerotic disease [34,35].

Another issue worthy of consideration is the occurrence of microembolic phenomena after carotid revascularization. New ischemic lesions (NILs) detected by diffusion-weighted MRI are frequently observed after carotid artery stenting (CAS) and mainly reflect periprocedural embolic events. Previous studies have demonstrated that the development of ipsilateral NILs after CAS is associated with multiple factors, including plaque-related characteristics such as intraplaque hemorrhage and lipid-rich necrotic core, as well as procedural factors including postdilation and stent-related features [36]. Although many of these lesions are clinically silent, their potential long-term prognostic and radiographic significance remains an important topic of investigation [37,38]. Because the present study focused on preoperative carotid HR-MRI phenotypes and long-term cardiovascular outcomes, systematic postprocedural brain MRI assessment was beyond the scope of this study. Future studies integrating preoperative plaque vulnerability, periprocedural embolic burden, and longitudinal brain imaging findings are warranted.

Several limitations should be acknowledged. First, this was a single-center retrospective study, and selection bias cannot be excluded. Second, the Silent High-Risk class had a relatively small sample size, which may affect the precision of risk estimates. Third, although the LCA indicators were selected based on biological plausibility and clinical relevance, fibrous cap thinning or rupture was not included in the present evaluation because fibrous cap status was not included as an independent variable in the predefined standardized image review protocol for this retrospective cohort. In addition, latent class solutions may vary across populations and require external validation. Fourth, post-procedural medical therapy, treatment adherence, and longitudinal changes in plaque morphology were not fully captured, which may influence long-term MACE risk. Finally, the primary endpoint was a composite of cardiovascular death, nonfatal myocardial infarction, coronary revascularization and stroke. Therefore, the mechanisms underlying individual event types may differ.

## 5. Conclusions

In patients undergoing carotid revascularization, latent class analysis identified three distinct clinical–radiological carotid vulnerability phenotypes: Systemic High-Risk, Silent High-Risk, and Stable/Moderate. Both high-risk phenotypes were associated with worse MACE-free survival compared with the Stable/Moderate phenotype. These findings suggest that integrated assessment of carotid plaque vulnerability, contralateral disease burden, and clinical risk status may improve long-term cardiovascular risk stratification after carotid revascularization. Further multicenter studies are warranted to validate these phenotypes and determine whether phenotype-guided surveillance and secondary prevention can improve clinical outcomes.

## Figures and Tables

**Table 1:** Acquisition parameters of the carotid HR-MRI protocol.

Parameters	3D TOF	Black-Blood T1WI	Black-Blood T2WI	SNAP	Contrast-Enhanced T1WI
TR, ms	20	800	4800	12	800
TE, ms	4	10	50	6.8	10
FOV, mm × mm	160 × 160	160 × 160	160 × 160	160 × 160	160 × 160
In-plane resolution, mm × mm	0.6 × 0.6	0.6 × 0.6	0.6 × 0.6	0.6 × 0.6	0.6 × 0.6
Slice thickness, mm	2	2	2	2	2
Number of slices	40	20	20	40	20

Note: HR-MRI = high-resolution carotid vessel-wall magnetic resonance imaging; 3D = three-dimensional; TOF = time-of-flight; T1WI = T1-weighted imaging; T2WI = T2-weighted imaging; SNAP = simultaneous non-contrast angiography and intraplaque hemorrhage; TR = repetition time; TE = echo time; FOV = field of view.

**Table 2:** Clinical and imaging characteristics of the study population.

Variables	Overall (*n* = 289)	Class 1 (*n* = 128)	Class 2 (*n* = 37)	Class 3 (*n* = 124)	*p*
Age, years	65.12 ± 9.46	66.77 ± 7.92	65.49 ± 7.06	63.31 ± 11.13	0.014
Male, *n* (%)	237 (82.0)	113 (88.3)	34 (91.9)	90 (72.6)	0.001
BMI (kg/m^2^)	25.10 ± 3.13	24.94 ± 2.90	25.62 ± 2.76	25.11 ± 3.44	0.540
Hypertension, *n* (%)	203 (70.2)	92 (71.9)	26 (70.3)	85 (68.5)	0.846
Diabetes, *n* (%)	112 (39.3)	51 (40.5)	17 (45.9)	44 (36.1)	0.524
Hyperlipidemia, *n* (%)	167 (69.3)	78 (72.2)	20 (58.8)	69 (69.7)	0.334
Symptoms, *n* (%)	216 (74.7)	128 (100.0)	0 (0.0)	88 (71.0)	<0.001
CVD history, *n* (%)	131 (45.3)	72 (56.2)	11 (29.7)	48 (38.7)	0.002
TC, mmol/L	3.92 ± 1.03	3.97 ± 1.01	3.75 ± 0.85	3.94 ± 1.09	0.599
HDL-c, mmol/L	1.08 ± 0.26	1.07 ± 0.25	1.04 ± 0.27	1.11 ± 0.28	0.341
LDL-c, mmol/L	2.20 ± 0.84	2.23 ± 0.85	2.02 ± 0.67	2.23 ± 0.87	0.440
TG, mmol/L	1.23 (0.91, 1.64)	1.25 (0.93, 1.69)	1.30 (1.04, 2.30)	1.17 (0.86, 1.55)	0.436
Procedure types, *n* (%)					<0.001
CEA	149 (51.6)	80 (62.5)	22 (59.5)	47 (37.9)	
CAS	140 (48.4)	48 (37.5)	15 (40.5)	77 (62.1)	
Carotid plaque					
Ipsilateral					
IPH	146 (50.5)	109 (85.2)	37 (100.0)	0 (0.0)	<0.001
LRNC	283 (97.9)	128 (100.0)	37 (100.0)	118 (95.2)	0.017
Ulceration	111 (38.4)	55 (43.0)	18 (48.6)	38 (30.6)	0.052
NWI	89.94 ± 8.20	91.18 ± 7.61	90.04 ± 5.65	88.63 ± 9.21	0.047
Contralateral					
IPH	76 (26.4)	58 (45.3)	15 (40.5)	3 (2.4)	<0.001
LRNC	164 (56.9)	85 (66.9)	25 (67.6)	54 (43.5)	<0.001
Ulceration	47 (16.3)	26 (20.3)	8 (21.6)	13 (10.6)	0.073
Moderate-to severe stenosis	102 (35.3)	59 (46.1)	13 (35.1)	30 (24.2)	0.001

Data are presented as mean ± standard deviation, median (interquartile range), or number (percentage), as appropriate. Class 1, Systemic High-Risk phenotype; Class 2, Silent High-Risk phenotype; Class 3, Stable/Moderate phenotype. BMI, body mass index; CVD, cardiovascular disease; TC, total cholesterol; HDL-c, high-density lipoprotein cholesterol; LDL-c, low-density lipoprotein cholesterol; TG, triglyceride; CEA, carotid endarterectomy; CAS, carotid artery stenting; IPH, intraplaque hemorrhage; LRNC, lipid-rich necrotic core; NWI, normalized wall index.

**Table 3:** Multivariable Cox regression for MACE, with Class 3 as the reference group.

Variable	HR	95% CI	*p*
Class 3: Stable/Moderate	reference		
Class 1: Systemic High-Risk	1.65	1.15–2.37	0.039
Class 2: Silent High-Risk	1.43	1.12–2.15	0.034
Age	1.01	0.98–1.04	0.465
Gender	1.86	0.84–4.14	0.127
Procedure types	1.20	0.72–1.99	0.483

## Data Availability

The datasets generated and analyzed during the current study are not publicly available due to institutional regulations and patient privacy restrictions but may be available from the corresponding author upon reasonable request and with appropriate institutional approval.

## Supplementary Materials

The following supporting information can be downloaded at: https://www.mdpi.com/article/10.3390/jcm15155884/s1, Table S1: Histopathological validation of carotid high-resolution MRI-derived plaque components. References [39,40,41] are cited in the Supplementary Materials.

## Abbreviations

The following abbreviations are used in this manuscript:

CEA	Carotid endarterectomy
CAS	Carotid artery stenting
LCA	Latent class analysis
BMI	Body mass index
CVD	Cardiovascular disease
HDL-c	High-density lipoprotein cholesterol
LDL-C	Low-density lipoprotein cholesterol
TC	Total cholesterol
TG	Triglycerides
IPH	Intraplaque hemorrhage
LRNC	Lipid-rich necrotic core
MACE	Major adverse cardiovascular events
HR-MRI	High-resolution magnetic resonance imaging
