# Supplementary material for: High-Resolution MRI-Based Clinical–Radiological Phenotypes of Carotid Vulnerability and Long-Term Outcomes After Carotid Revascularization: A Single-Center Retrospective Cohort Study
Source: J Clin Med. 2026 Jul 28;15(15):5884. doi: 10.3390/jcm15155884 (PMC13467100; doi:10.3390/jcm15155884)
Supplement: Supplementary file 1 [file jcm-15-05884-s001.zip › jcm-4436390-supplementary.pdf]

Supplementary Table S1. Histopathological validation of carotid high-resolution MRI-derived plaque components

**MRI-derived feature**	**MRI characteristics**	**Histopathological correlate**	**Representative studies**
Intraplaque hemorrhage (IPH)	Hyperintensity on T1-weighted imaging and SNAP	Intraplaque blood products, particularly methemoglobin accumulation	Hatsukami et al. [1]; Yuan et al. [2]
Lipid-rich necrotic core (LRNC)	Characterized by specific signal patterns on multi-contrast vessel-wall MRI	Lipid accumulation and necrotic tissue within the plaque core	Yuan et al. [2]; Saam et al. [3]
Fibrous cap thinning/rupture	Surface irregularity, disruption, or loss of the fibrous cap signal	Thinning or disruption of the collagen-rich fibrous cap	Hatsukami et al. [1]

[1] Hatsukami TS, Ross R, Polissar NL, Yuan C. Visualization of fibrous cap thickness and rupture in human atherosclerotic carotid plaque in vivo with high-resolution magnetic resonance imaging.Circulation. 2000;102(9):959-964.

[2] Yuan C, Mitsumori LM, Ferguson MS, Polissar NL, Echelard D, Ortiz G, Small R, Davies JW, Kerwin WS, Hatsukami TS. In vivo accuracy of multispectral magnetic resonance imaging for identifying lipid-rich necrotic cores and intraplaque hemorrhage in advanced human carotid plaques. Circulation. 2001;104(17):2051-2056.

[3] Saam T, Ferguson MS, Yarnykh VL, Takaya N, Xu D, Polissar NL, Hatsukami TS, Yuan C. Quantitative evaluation of carotid plaque composition by in vivo MRI. Arteriosclerosis, Thrombosis, and Vascular Biology. 2005;25(1):234-239.
